# Scrotum Pain Leading to the Diagnosis of a Compression Fracture

**DOI:** 10.7759/cureus.77207

**Published:** 2025-01-09

**Authors:** Shuji Toriumi, Ryosuke Maruiwa, Minoru Takemoto

**Affiliations:** 1 Department of Diabetes, Metabolism and Endocrinology, International University of Health and Welfare, Narita Hospital, Narita, JPN; 2 Department of Orthopedics, International University of Health and Welfare, Narita Hospital, Narita, JPN

**Keywords:** compression fracture, differential diagnoses, emergency, prompt imaging, scrotal pain

## Abstract

A 45-year-old male presented to the emergency department with an acute onset of severe lumbosacral pain and scrotum pain. Despite initial differential diagnoses, including testicular torsion, perineal necrotizing fasciitis, and acute epididymitis, subsequent imaging revealed a burst fracture of the 12th thoracic vertebra. This case highlights the importance of considering vertebral compression fractures in the differential diagnosis of acute scrotum pain, especially in patients with atypical presentations.

## Introduction

Testicular pain can originate from the testicle itself or surrounding tissues. Primary testicular causes include orchitis and testicular rupture due to trauma, while secondary causes involve inflammation of the epididymis, a structure attached to the upper part of the testicle, and impaired testicular blood flow due to conditions such as varicocele and testicular torsion [1,2]. Regardless of the cause, many diseases associated with testicular pain require proper evaluation and specialized treatment [3,4]. In particular, pain caused by impaired blood flow, such as testicular torsion or varicocele, necessitates urgent diagnosis and intervention within six to eight hours after onset [1].

Additionally, aneurysms have been reported as a cause unrelated to the testicle itself [5]. In this case report, we describe a patient who presented to the emergency department with severe testicular pain but showed no abnormalities in the testicle or surrounding tissues. Computer tomography (CT) and magnetic resonance imaging (MRI) revealed a spinal compression fracture, suggesting that inflammation associated with the fracture was the cause of the testicular pain. There have been few previous reports of severe testicular pain caused by a spinal compression fracture [6]. This case highlights the importance of considering spinal compression fractures as a differential diagnosis for severe testicular pain.

## Case presentation

A 45-year-old man presented to the emergency department with a sudden onset of severe lumbosacral and scrotal pain, where even the slightest touch to the scrotum was excruciating. His medical history included head trauma at three years of age requiring surgery, childhood epilepsy, and urinary calculi. The patient's last remembered epileptic seizure was five to six years ago, but they have not been taking anti-epileptic drugs recently. The patient was also not taking any regular medications. He had no allergies. During physical examination, the patient was alert and oriented, with normal vital signs. Abdominal examination revealed tenderness and rebound tenderness, with marked spontaneous pain in both scrota. No edema or paralysis was observed in the lower extremities.

The laboratory findings at the time of the emergency department presentation are shown in Table 1.

**Table 1:** Laboratory findings at admission Laboratory findings at the time of admission demonstrated leukocytosis without concomitant elevation of inflammatory markers, such as C-reactive protein. Urinary examination was unremarkable.

Complete blood count	Patient values	Units
WBC	22,270	μL
RBC	562x10^4^	μL
Hb	16.4	g/dL
Plt	31x10^4^	μL
Coagulation		
PT	9.3	seconds
PT％	104	％
PT INR	0.95	
APTT	24.1	seconds
Biochemistry		
Alb	4.9	g/dL
AST	29	U/L
ALT	30	U/L
GGT	28	U/L
Alp	64	U/L
BUN	14.4	mg/dL
Cre	1.17	mg/dL
CRP	0.07	mg/dL
CK	198	U/L
Na	140	mEq/L
K	3.4	mEq/L
Cl	＜106	mEq/L
Ca	9.7	mg/dL
Mg		mg/dL
Blood gas analysis (venous blood)		
pH	7.336	
pCO2	45.4	mmHg
pO2	28.4	mmHg
HCO3	24.3	mmol/L
Urinalysis		
prot	-	
sugar	-	
Occult blood	±	
Keton	-	

**Figure 1:**
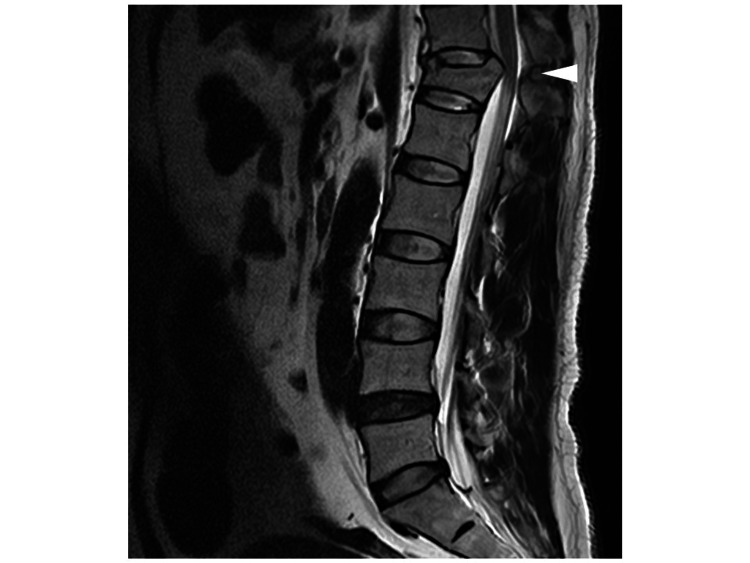
A spinal cord magnetic resonance T2-weighted image showing a burst fracture of the 12th thoracic vertebra The arrowhead indicates a burst fracture.

There was no history of trauma, and no obvious wounds were found around the testes, ruling out trauma as a cause. Ultrasound examination showed no decreased blood flow to the testes, ruling out testicular torsion. The ultrasound findings also ruled out varicocele. There was no history of mumps, and the patient had no fever at the time of presentation. Urine analysis showed no increase in white blood cells, ruling out orchitis and epididymitis. Testicular tumors were also ruled out based on ultrasound and physical examination findings. Further imaging with contrast-enhanced CT to investigate the cause of the testicular pain ruled out an abdominal aortic aneurysm but suggested a spinal compression fracture. As the patient developed bladder and rectal dysfunction, an MRI was performed. Spinal cord magnetic resonance T2-weighted revealed a burst fracture with compression of the 12th thoracic vertebra (Figure 1). Then, emergency surgery was performed. Postoperatively, the patient experienced residual numbness in the anus and left costal margin, but the scrotal pain improved.

## Discussion

The differential diagnoses of acute testicular pain include testicular torsion, perineal necrotizing fasciitis, acute epididymitis, varicocele, and chronic orchialgia [1,2]. Isolated testicular pain may be an unusual clinical presentation of a symptomatic abdominal aortic aneurysm [5]. Given the wide range of life-threatening conditions that can manifest as acute scrotal pain, differential diagnosis is of paramount importance. In patients experiencing idiopathic scrotal pain, the S2-S4 spinal segments and nerve roots are usually involved, and the causative lesion is located at the L1 level or below [6,7]. Recently, it has been reported that spontaneous spinal subdural hematoma at L1-L3 caused by COVID-19 infection showed acute testicular pain [8].

In this case, the scrotal pain is likely attributed to the spread of inflammation from the burst fracture of the 12th thoracic vertebra. Thoracolumbar spine fractures, encompassing vertebrae T11 to L2, constitute the most common type of spinal fracture, accounting for 90% of all cases. This region, recognized as the weakest segment of the spine from a biomechanical standpoint, is particularly susceptible to injury [9,10]. High-energy trauma is the primary cause in younger individuals, while low-energy trauma, such as falls, is more common in older populations. A significant proportion of thoracolumbar spine fractures, ranging from 20% to 40%, are associated with neurological complications.

In our case, there was no recent trauma. Bone mineral density assessment upon admission showed a lumbar spine T-score of -2.0% (young adult mean (YAM) value of 73%) and left femoral T-score of 91% (YAM value of 91%). Meanwhile, the patient's lumbar spine T-score (-2.0%) and YAM value (73%) upon admission did not meet the diagnostic criteria for osteoporosis, indicating the presence of osteopenia. Although the serum calcium level upon admission was within the normal range, serum phosphorus and PTH levels were not measured, and the possibility of hyperparathyroidism, which might be related to osteopenia, cannot be completely ruled out.

The exact reason for the patient's spinal compression fractures remains unclear. However, his past medical history included head trauma at age three requiring surgery and childhood epilepsy. A head CT scan taken upon admission showed signs of cerebral contusion and craniotomy scars in the left parietal lobe. Spike waves were also observed in the same area on EEG. The patient's last remembered epileptic seizure was five to six years ago, but he had not been taking anti-epileptic drugs recently. Therefore, there is a possibility that he experienced a nocturnal seizure, leading to a compression fracture.

It is well known that patients with generalized tonic-clonic seizures have an increased trauma risk, which could have led to spinal compression fractures [11,12]. Therefore, it is likely that his compression fractures were caused by tonic-clonic seizures in the presence of spinal osteopenia.

## Conclusions

This case report emphasizes the importance of considering vertebral compression fractures in the differential diagnosis of patients presenting with acute testicular pain, even in the absence of typical symptoms. Prompt imaging studies are essential for accurate diagnosis and timely management.
